# Complete Genome Sequence of *Pseudomonas psychrotolerans* CS51, a Plant Growth-Promoting Bacterium, Under Heavy Metal Stress Conditions

**DOI:** 10.3390/microorganisms8030382

**Published:** 2020-03-09

**Authors:** Sang-Mo Kang, Sajjad Asaf, Abdul Latif Khan, Lubna, Adil Khan, Bong-Gyu Mun, Muhammad Aaqil Khan, Humaira Gul, In-Jung Lee

**Affiliations:** 1 School of Applied Biosciences, Kyungpook National University, Daegu 41566, Korea; kmoya@hanmail.net (S.-M.K.); mun0301@naver.com (B.-G.M.); aqil_bacha@yahoo.com (M.A.K.); 2 Natural and Medical Sciences Research Center, University of Nizwa, Nizwa 616, Oman; sajadasif2000@gmail.com (S.A.); latifepm78@yahoo.co.uk (A.L.K.); adilsafi122333@gmail.com (A.K.); 3 Department of Botany, Garden Campus, Abdul Wali Khan University Mardan, Mardan 23200, Pakistan; lubnabilal68@gmail.com (L.); gulhumaira@awkum.edu.pk (H.G.)

**Keywords:** *Pseudomonas psychrotolerans*, genome sequencing, plant growth promotion, gibberellin biosynthesis, heavy metal resistance, pan-genome

## Abstract

In the current study, we aimed to elucidate the plant growth-promoting characteristics of *Pseudomonas psychrotolerans* CS51 under heavy metal stress conditions (Zn, Cu, and Cd) and determine the genetic makeup of the CS51 genome using the single-molecule real-time (SMRT) sequencing technology of Pacific Biosciences. The results revealed that inoculation with CS51 induced endogenous indole-3-acetic acid (IAA) and gibberellins (GAs), which significantly enhanced cucumber growth (root shoot length) and increased the heavy metal tolerance of cucumber plants. Moreover, genomic analysis revealed that the CS51 genome consisted of a circular chromosome of 5,364,174 base pairs with an average G+C content of 64.71%. There were around 4774 predicted protein-coding sequences (CDSs) in 4859 genes, 15 rRNA genes, and 67 tRNA genes. Around 3950 protein-coding genes with function prediction and 733 genes without function prediction were identified. Furthermore, functional analyses predicted that the CS51 genome could encode genes required for auxin biosynthesis, nitrate and nitrite ammonification, the phosphate-specific transport system, and the sulfate transport system, which are beneficial for plant growth promotion. The heavy metal resistance of CS51 was confirmed by the presence of genes responsible for cobalt-zinc-cadmium resistance, nickel transport, and copper homeostasis in the CS51 genome. The extrapolation of the curve showed that the core genome contained a minimum of 2122 genes (95% confidence interval = 2034.24 to 2080.215). Our findings indicated that the genome sequence of CS51 may be used as an eco-friendly bioresource to promote plant growth in heavy metal-contaminated areas.

## 1. Introduction

Heavy metal stress greatly decreases crop growth and productivity. It is known as a major threat in various terrestrial ecosystems worldwide [1]. Currently, extensive industrialization directly increases the accumulation of heavy metals in soils, which consequently has detrimental effects on crop growth and productivity [2]. Although several heavy metals (e.g., Mn, Cu, Co, Zn, Mo, and Ni) are vital for important biological processes [1,3], the excessive accumulation of these heavy metals with other highly toxic heavy metals including Pb, Cd, As, Hg, Cr, and Al can limit crop productivity [4]. The accumulation of heavy metals in soils directly affects the texture and pH of the soil, which ultimately may reduce the growth of plants by exerting detrimental effects on various biological processes in plants [5]. 

Several studies have reported the positive role of microorganisms in plant growth and productivity in heavy metal-contaminated soils [6,7]. Plant growth-promoting rhizobacteria (PGPR) are a group of bacteria that can improve plant growth and productivity [8]. These microorganisms are found predominantly in the rhizosphere. In the literature, PGPR have been reported to alleviate heavy metal stress through several mechanisms such as mobilization, immobilization, and heavy metal transformation [9]. Furthermore, various PGPR present in the soil around plant roots can facilitate plant growth either by producing various plant growth regulators or providing and facilitating nutrients uptake [10]. PGPR could improve plants tolerance to different abiotic stresses, including heavy metal stress [11]. Several genera of heavy metal-tolerant bacteria, including *Pseudomonas*, *Bacillus*, *Methylobacterium*, and *Streptomyces* have the ability to increase the growth and production of crops by minimizing the negative effects of heavy metal stress [12]. In the last decade, various genera of PGPR including *Pseudomonas*, *Bacillus*, *Rhizobium, Pantoea*, *Burkholderia*, *Paenibacillus*, *Enterobacter*, *Azospirillum*, *Achromobacter*, *Methylobacterium*, *Variovorax*, and *Microbacterium* have been found to play a role in tolerance to abiotic stress [13,14]. 

Among the various PGPR genera, the genus *Pseudomonas* has attracted attention due to several unique characteristics. The genus *Pseudomonas* has a wide distribution, and it is very easy to culture under laboratory conditions [15]. *Pseudomonas* can be found in various environments, such as plants [16], straw [16], soil [17], animals [18], and saline water [19,20]. Currently, 322 valid species names have been reported in the literature (http://www.bacterio.net). The beneficial role of *Pseudomonas* has been described in various studies [21,22]. To further understand the traits of *Pseudomonas* at the genetic level, whole genome sequence analysis is widely used, and various species from the genus have been studied genetically [23,24]. 

*P. psychrotolerans* is a diverse species predominant in nature [25]. It was initially isolated from animals in 2004 [26]. The first whole genome sequence was released in 2012 from a *P. psychrotolerans* strain isolated from copper coins [27]. In addition, other studies have reported the whole genome sequences of *P. psychrotolerans* strains isolated from a clinical sample [28]. In the case of plants, Adorada, et al. [29] reported the whole genome sequences of *P. psychrotolerans* isolated from diseased rice. Furthermore, Xie et al. [30] characterized several *Pseudomonas* spp., including *P. psychrotolerans* PRS08-11306, from rice seeds.

The plant growth-promoting activities of various *P. psychrotolerans* strains have been reported previously; however, their plant growth-promoting characteristics have not been fully explored [22,31]. Similarly, *P*. *psychrotolerans* was reported for silicate solubilization and improve soybean plant growth [32]. Therefore, the identification of the plant growth-promoting characteristics of the current strain is crucial. In the current study, we investigated the CS51 whole genome sequence to determine plant growth-promoting characteristics of *P. psychrotolerans*. The findings of this study could help elucidate the complex biological mechanisms of the current strain responsible for plant growth and tolerance to heavy metal stress. The whole genome sequencing of CS51 would contribute to the investigation of plant growth-promoting activity and tolerance characteristics against different environmental stresses. In addition, unique traits shared among already sequenced *Pseudomonas* species may be identified. Hence, it will offer insight into the evolutionary changes that have occurred within this genus. 

## 2. Material and Methods

### 2.1. Study Site and Microbe Isolation 

Soil samples were collected from an agriculture field in Gyeongbuk province, and microbes were isolated. First, the soil was diluted, and it was plated on nutrient agar plates. Based on the morphology, different colonies were selected, which were then further purified by streaking. For each colony, the morphology and texture were determined and recorded. Finally, the colonies were randomly selected for further analyses; the selected colonies were maintained on a nutrient agar slant at 4 °C.

### 2.2. Solubilization of Insoluble Phosphate

The rhizospheric bacteria were assessed for potential phosphate solubilization on Pikovskaya’s (PVK) agar medium (supplemented with 1.5% Bacto Agar), as described previously [33]. The *rhizospheric* bacterial colonies were stabbed in triplicate on PVK agar plates using sterile toothpicks. The plates were incubated at 28 °C. Bacterial growth was evaluated every 24 h for 7 days after incubation, both the halo and colony diameters were measured. To measure the diameter of the halo zone, the colony diameter was subtracted from the halo. The data are the mean of three experiments.

### 2.3. Screening for Indole-3-Acetic Acid (IAA) and Gibberellins (GAs) in Cell-Free Cultures

The broth culture of the bacteria was centrifuged at 2500× *g* for 10 min at 4 °C to separate the cells from the supernatant. Subsequently, 0.45 μm cellulose acetate filters (DISMIC^®^; Frisenette ApS, Knebel, Denmark) were used to remove debris from the resulting supernatant, and the clear supernatant was used for IAA analysis. Initially, Salkowski’s reagent was used for the confirmation of IAA production [34]. The bacterial supernatant (2 mL) was mixed with 1 mL of Salkowski’s reagent (50 mL 35% HClO_4_, 1 mL 0.5 M FeCl_3_) and was placed for 30 min in the dark. After incubation, the absorbance was taken at 530 nm (pink color) (T60 UV-VIS Spectrophotometer; PG Instruments, Leicester, UK) and quantified using the calibration curve of an IAA standard with linear regression analysis.

The IAA in bacterial cell-free cultures (5 mL) was quantified by GC-MS/SIM (6890 N Network GC System and 5973 Network Mass Selective Detector; Agilent Technologies, Palo Alto, CA, USA) as described previously [35]. Similarly, the quantification of GAs in the CS51 culture was carried out according to a previous protocol [36]. Bacterial culture filtrates supplemented with [^2^H_2_] GA standards were processed for the detection, identification, and quantification of GAs by gas chromatography and mass spectroscopy.

### 2.4. Screening of Endophytes on GA-Deficient Waito-C Dwarf Rice

To examine the ability of the isolated bacteria for GA production, *Waito-C,* in which the biosynthesis pathway for GA is suppressed, was used. Initially, the seeds were treated with 2.5% sodium hypochlorite (NaClO) for 30 min to sterilize the surface, followed by washing with autoclave-distilled water three times. The seeds were incubated with 20 mg/L of uniconazole for 24 h to detect GA production. GA biosynthesis was arrested by treating the seeds with uniconazole to determine the effects of CS51 on mutant rice. As reported previously, pre-germinated *Waito-C* seeds were transferred to autoclaved pots [37]. The root and shoot lengths were calculated.

### 2.5. Screening of Bacteria for Heavy Metal Stress and Germination of Seeds

Initially, *P. psychrotolerans* CS51 was screened for resistance to three heavy metals (copper, zinc, and cadmium). *P. psychrotolerans* CS51 had a higher resistance to Zn and Cd compared with Cu. The bacteria were grown on plates with different concentrations (1 mM and 10 mM) of Cu, Zn, and Cd and were incubated at 28 °C. Bacterial growth was evaluated every 24 h for 7 days. The bacterial strains were streaked on plates in triplicate.

### 2.6. Plant–Microbe Interaction under Heavy Metal Stress

In the current study, cucumber seeds were used. The surface of approximately 100 seeds was sterilized by soaking with 75% ethanol for 2 min. Subsequently, the seeds were treated with 1% NaClO for 1 min. The seeds were then washed repeatedly with autoclave-distilled water to completely remove any residual NaClO. The seeds were placed on a pre-soaked filter paper containing 1.5 mL autoclave-distilled water in a dark environment. After 3 days, germinated seeds with equal lengths were transplanted into sterile plastic pots (22 × 15 × 7 cm). Three pots were used per replication. The pots were placed in a growth chamber. The conditions of the growth chamber were as follows: 25 ± 2 °C with a 12 h light/dark cycle and a relative humidity of 60%. At the 2 leaves stage, morphologically similar samples were selected for further analysis. 

*P. psychrotolerans* CS51 was cultured in LB broth at 30 °C for 3 days in a shaking incubator at 200 rpm. The resulting bacterial cultures were centrifuged at 10,000× *g* for 10 min, and the supernatant was diluted 10 times; 50 mL of the diluted supernatant was applied to seedlings at the 2 leaves stage. The treatment conditions were as follows: (1) Control cucumber seedling without microbial cells; (2) cucumber seedling with CS51; (3) 5 mM Cu, CS51 + 5 mM Cu, 10 mM Cu, and CS51 + 10 mM Cu; (4) 5 mM Zn, CS51 + 5 mM Zn, 10 mM Zn, and CS51 + 10 mM Zn. During the experiment, the plants were exposed to the following environmental conditions: 14/10 h day/night cycle at 28/25 °C with 60–70% relative humidity. The control plant was grown only in water, and a second control was treated with bacterial cell-free culture. For statistical analysis, three plants were randomly selected. The treated and negative control samples were compared. At this stage, the root and shoot lengths of the plants were measured. Chlorophyll contents were recorded for fully expanded leaves using a chlorophyll meter. After harvesting, the fresh weight was measured immediately. To measure the dry weight, the plants were first placed in an oven at 70 °C for 72 h.

### 2.7. DNA Extraction, Genome Sequencing, and Genome Assembly

Qiagen™ QIAamp DNA Mini Kit (Qiagen, Hilden, Germany) was used for the extraction of genomic DNA from the overnight cell suspension cultures of CS51 bacteria for whole genome sequencing. As described previously, the sequencing of the complete genome was performed using the single-molecule real-time (SMRT) sequencing technology of Pacific Biosciences (PacBio, Menlo Park, CA, USA) [38]. In brief, from high-molecular-weight genomic DNA (120.0 ng/μL), a PacBio large-insert library (15–20 kb) was generated, and V2 SMRT cells were used for sequencing by P4-C2 chemistry with a running movie for 4 h at the Duke Center for Genome and Computational Biology, Duke University (Durham, NC, USA). The PacBio-generated data file was in the HDF5 format (*.h5), and the corresponding input file of SMRT Analysis software was a bas.h5 file or an associated bax.h5 file. QUAST 2.3 was used to ensure the data quality of the assemblies [39]. A total of 96,384 reads were generated with a mean read length of 13,888 base pairs. The total reads were de novo assembled into the form of a circular chromosome with an average genomic coverage of 150.26 reads using the Hierarchical Genome Assembly Process (HGAP) workflow in SMRT Portal (version 2.1.1). 

### 2.8. Genome Annotation

The NCBI Prokaryotic Genome Annotation Pipeline was used to perform complete genome annotation [40]. The coding genes were predicted using an ab initio gene prediction algorithm with homology-based methods. Furthermore, from the annotation process, functional genomic units such as structural RNAs (23S, 5S, and 16S), tRNAs, and small noncoding RNAs were determined. Rapid Annotation using Subsystem Technology (RAST) version 3.0 and the Integrated Microbial Genomes (IMG) platform were used to perform additional coding gene prediction and functional annotation [41,42,43,44]. The assembled and annotated sequences of CS51were submitted to GenBank with accession number CP021645. 

### 2.9. Comparative Genome Analysis

To elucidate the genomic characteristics of *P. psychrotolerans* CS51, comparative assessments were performed with the recently reported genome sequences of *P. aeruginosa*, *P. psychrotolerans* PRS08, *P. putida*, and *P. syringae*, all of which were retrieved from the NCBI database. Gene prediction and functional annotation of CS51 were performed using the RAST subsystem [41,42,43]. A circular genomic map of each genome was generated by BioCircos using EDGAR version 2.0 for comparison [45]. Each circular genomic map was generated with BLAST+ using standard parameters (70% lower and 90% upper cutoff for identity, *E*-value of 10) with the CS51 genome as the “alignment reference genome”. Similarly, pan-genome and core genome analyses of CS51 against related species were carried out using EDGAR version 2.0 [45] and PGAP version 1.12 [46]. 

## 3. Results and Discussion

### 3.1. Isolation of PGPR from Soil

**Figure 1:**
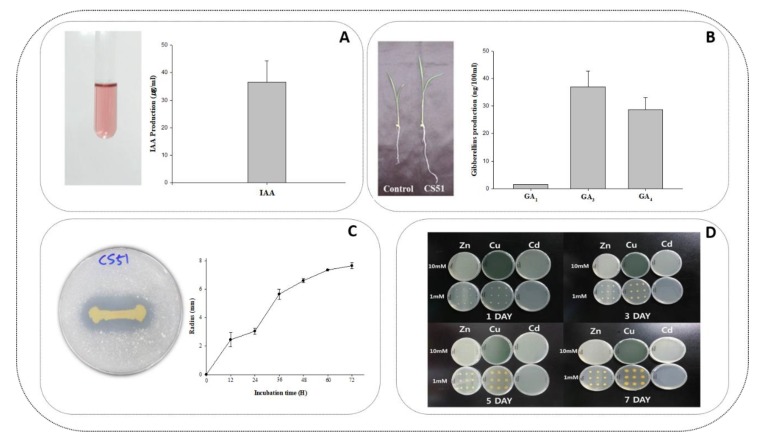
(**A**) Salkowski test for indole-3-acetic acid (IAA) determination and the amount of IAA produced by CS51 by GC-MS/SIM spectrometry analysis, (**B**) growth promoting effect of CS51 inoculation on *Waito-C* (gibberellins (GA) deficient) rice plants and the amount of different GAs produced by CS51 by GC-MS/SIM spectrometry analysis, (**C**) growth pattern of CS51 in 1 mM and 10 mM Zn-supplemented, Cu-supplemented, and Cd-supplemented media, (**D**) phosphate-solubilizing activity of CS51 on national botanical research institute’s phosphate (NBRIP) growth media plates, and incubated for 7 days at 30°C. The clarification halos show P solubilization and the maximum size of the clarification halos was reached after 72 values given are means of three replicates. Error bars indicate standard deviations.

Initially, around 10 bacterial strains were isolated from soil samples (associated with the roots of the plants). All of the bacterial strains were analyzed based on morphological characteristics, such as the shape of the colony, growth on the medium, growth rate and pattern, base color, margin characteristics, and texture, which revealed the same morphotypes. However, all strains were initially screened for heavy metals resistance and the production of growth-promoting phytohormones such as IAA and GAs in their culture filtrate (Figure 1A,B). Among all the PGPR strains, *P. psychrotolerans* CS51 was selected for further analysis, as it produced GAs and IAA and exhibited high resistance to heavy metals such as Zn and Cu (Figure 1A–C). Previously, *P*. *psychrotolerans* CS51 was reported for silicate solubilizing and to improve soybean plant growth and development at paddy soil [32]. Similarly, one other strain *psychrotolerans* PRS08-11306 was also reported as a plant growth-promoting bacterium [22].

### 3.2. GA Production and Phosphate Solubilization Potential of P. psychrotolerans CS51 

The *P. psychrotolerans* CS51 culture filtrate was evaluated to determine its ability to secrete GAs in the growth medium. Different GAs (physiologically active and nonactive) were observed in the culture filtrate using a GC/MS selected ion monitor. The most predominantly detected GAs were GA_3_ (35 ng/100 mL), GA_4_ (28 ng/100 mL), and GA_1_ (3 ng/100 mL) in the different HPLC fractions. Among the different bioactive GAs, the concentration of GA_3_ and GA_4_ was significantly higher than that of other GAs (Figure 1B). In addition, the culture filtrate was analyzed for the presence of IAA (Figure 1A). The level of IAA was 35 ng/mL. A number of plant growth-promoting bacteria have been reported to produce GAs and IAA previously by various researchers [47,48,49,50]. The ability of CS51 to produce IAA and GAs (Figure 1AB) was similar to previously reported plant growth promoting bacterial species [51,52,53]. Previous research revealed that the production of IAA and GAs is considered the most important attribute of plant growth-promoting bacteria [37,47]. Furthermore, the bacteria were assessed for their ability to solubilize inorganic phosphate. The formation of a clear zone around the colony indicated the phosphate-solubilizing ability of the CS51 bacteria (Figure 1D). Previous studies have reported that different endophytic *Pseudomonas* species can produce GAs and insoluble phosphates [54,55]. Furthermore, CS51 has demonstrated phosphate-solubilizing ability, which is considered one of the most crucial abilities of plant growth-promoting bacteria [55,56]. Phosphate-solubilizing bacteria may enhance the uptake of phosphorus by converting inorganic phosphorus to a more available form [57]. Phosphate solubilization is mainly associated with the production of microbial metabolites, including organic acids, which can decrease the pH of the culture media [56,58]. Phosphate-solubilizing microbial populations are present in soils, and the microbes may be used as biofertilizers for crop production and would be beneficial for sustainable agriculture.

### 3.3. Promotion of Cucumber Plant Growth by P. Psychrotolerans CS51 under Heavy Metal Stress

The use of metal-resistant plant growth-promoting bacteria constitutes an important technology for enhancing biomass production as well as the tolerance of plants to heavy metals. In the present study, *P. psychrotolerans* CS51 was initially grown on culture plates with various heavy metals (Zn, Cu, and Cd). CS51 exhibited high resistance to Zn and Cu compared with Cd (Figure 1C). In order to examine its contribution to plant growth, isolates of CS51 were used for in vivo experiments. Based on the initial selection, we only used the heavy metals Zn and Cu to investigate the potential of CS51 PGPR in attenuating heavy metal effects. 

**Figure 2:**
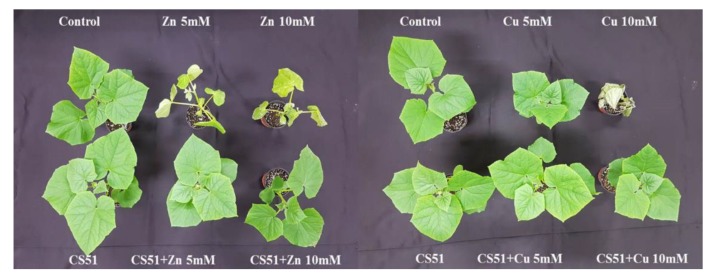
Effect of CS51 on the growth of cucumber plant, under control conditions, copper (5 mM and 10 mM Cu) and zinc (5 mM and 10 mM Zn) stress. Control, untreated plants; CS51, *P. psychrotolerans*-treated; Cu 5 mM and Cu 10 mM, copper treated plants; Zn 5 mM and Zn 10 mM, zinc treated plants; Cu 5 mM and Cu 10 mM + CS51, copper + CS51-treated plants; Zn 5 mM and Zn 10 mM + CS51, zinc + CS51-treated plants.

**Figure 3:**
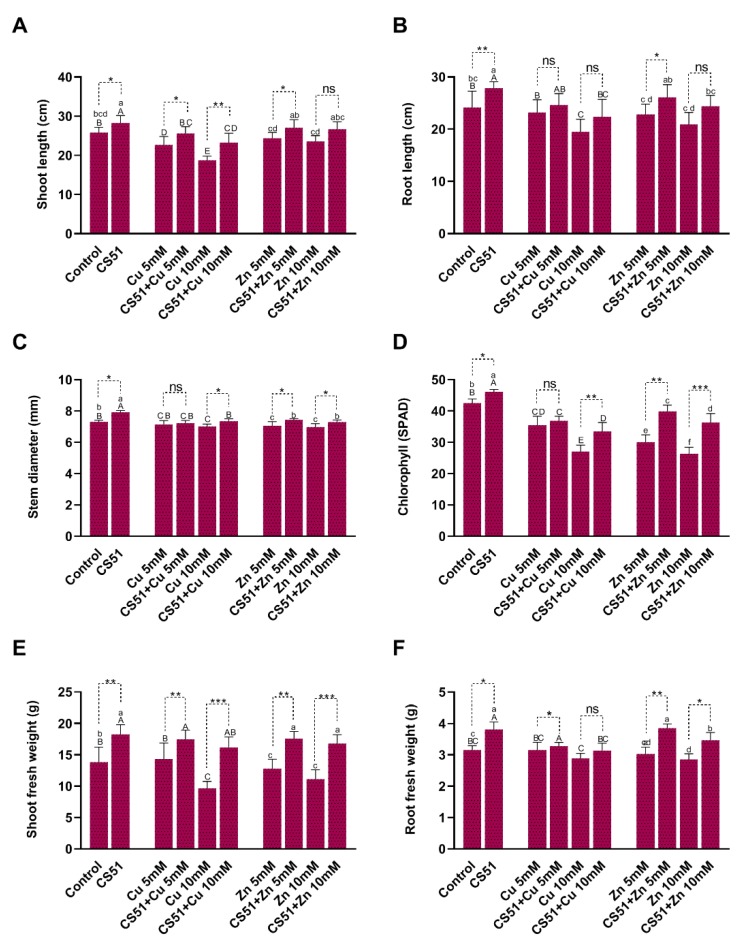
Effect of CS51 on the growth of cucumber plant growth, under control conditions, Cu (5 mM and 10 mM Cu), and Zn (5 mM and 10 mM Zn) stress. Growth parameters include (**A**) shoot length, (**B**) root length, (**C**) stem diameter, (**D**) chlorophyll contents (SPAD), (**E**) shoot fresh weight, and (**F**) root fresh weight. Data are means of five replicates along with standard error bars. Mean bars labeled with different letters (Capital letters = Control vs. Cu-stress; Small letters = Control vs. Zn-stress) are significantly different as evaluated by Duncan multiple range test (DMRT) analysis. ns, *, **, *** indicates non-significance or significance at 5%, 1%, and 0.1% probability levels, respectively. Data were analyzed with an unpaired student t test at 95% confidence intervals in cucumber plants treated with and without CS51 under both Cu and Zn stress.

Inoculation with CS51 increased the length of the shoot and root of cucumber plants. Under Cu and Zn (5 mM and 10 mM) stress, the shoot and root lengths significantly decreased in both inoculated and non-inoculated plants compared with control plants (Figure 2 and Figure 3A,B). The shoot and root lengths of the CS51-treated plants were 28.24 ± 1.90 cm and 27.86 ± 1.24 cm, respectively, compared with those of the control plants (25.78 ± 1.37 cm and 24.14 ± 3.14 cm, respectively) (Figure 2 and Figure 3A,B). Similarly, CS51 application increased the stem diameter under normal and heavy metal stress conditions (Figure 2 and Figure 3C). The Soil Plant Analysis Development (SPAD) value indicated that the chlorophyll content was significantly higher in cucumber plants inoculated with CS51 than in non-inoculated control plants (46.16 ± 0.74 cm and 42.54 ± 1.28 cm, respectively) (Figure 2 and Figure 3D). Similarly, there was a significant increase in shoot and root fresh weight (18.25 ± 1.55 and 3.81 ± 0.24, respectively) of the inoculated (with CS51) plants compared with the control (13.82 ± 2.42 and 3.16 ± 0.13 respectively) (Figure 2 and Figure 3A,B). Hence, the results reveal that the application of CS51 promote plant growth under normal and heavy metals stress conditions. Previously, several strains of bacteria have been reported to improve plant growth by regulating endogenous hormones [35,53,59,60]; however, there is still a need to identify novel strains. Several studies have demonstrated that metal-tolerant rhizobacteria can stimulate plant growth in the presence or absence of toxic concentrations of heavy metals, such as Ni [61], Cd [62], Pb [63], and Cr (VI) [64,65]. Kang et al. [59] reported that the inoculation of *Brassica rapa* with *Burkholderia cepacia* could increase the tolerance to Zn^+^ toxicity. The results of the current study indicated that the inoculation of plants with CS51 increased heavy metal tolerance, improving plant growth parameters.

### 3.4. General Genomic Features of P. psychrotolerans CS51

**Figure 4:**
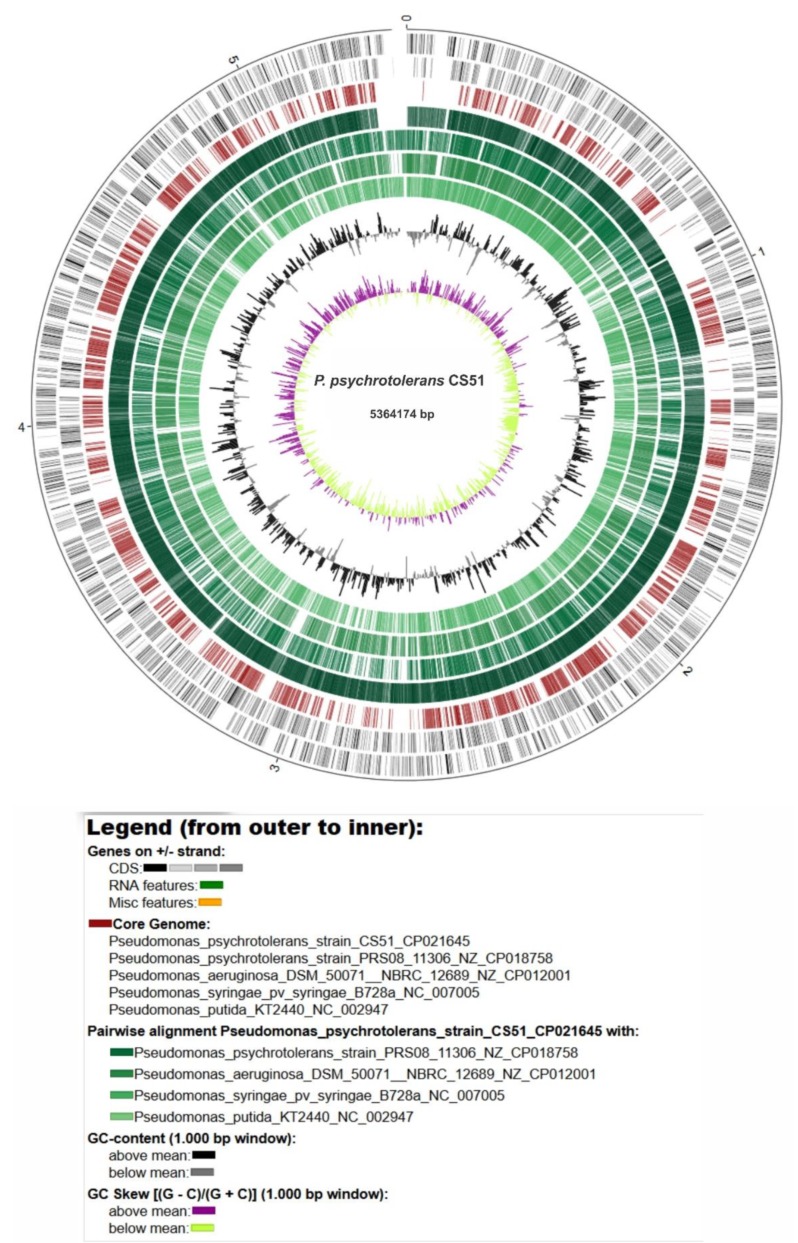
Circular representation of the *P. psychrotolerans* CS51 genome and comparison with related species. The green color intensity shows the pairwise alignment of *P. psychrotolerans* CS51 with related species. The inner two circles show the G+C content and skew.

The genome of the *P. psychrotolerans* CS51 strain consisted of a circular chromosome of 5,364,174 base pairs with an average G+C content of 64.71% (Figure 4). Previously, the similar genome sizes of different *Pseudomonas* species isolated from the endosphere and rhizosphere have been reported [66]. There were around 4774 predicted protein-coding sequences (CDSs) in 4859 genes, 15 rRNA genes, and 67 tRNA genes (Table 1). Around 3950 protein-coding genes with function prediction and 733 genes without function prediction were identified. Proteins, rRNAs, and tRNAs were encoded by 82.81%, 0.31%, and 1.40% of the complete genome, respectively. Among the predicted CDSs, around 3,683 CDSs (77.21%) could be assigned to the Clusters of Orthologous Groups (COG) database. The major COG categories were amino acid transport and metabolism (10.93%), general function and prediction only (7.98%), transcription (7.98%), and signal transduction and mechanisms (6.71%) (Figure S1). Similarly, the number of CDSs assigned to the Kyoto Encyclopedia of Genes and Genomes (KEGG) database was 1577, and the major pathways were global and overview maps (26.15%), amino acid metabolism (8.66%), carbohydrate metabolism (8.5%), and membrane transport (7.94%) (Figure S2).

### 3.5. Plant Growth-Promoting Potential of P. psychrotolerans CS51

Various species of bacteria have been reported to promote plant growth by providing phytohormones or enzymes [55,67,68]. The current study revealed that CS51 promoted plant growth through the production of GAs and IAA (Figure 1A,B). Furthermore, we identified genes related to auxin biosynthesis, which included tryptophan synthase beta chain (*TSb*) (CCZ28_16130), tryptophan synthase alpha chain (*TSa*) (CCZ28_16125), phosphoribosylanthranilate isomerase (PRAI), and anthranilate phosphoribosyltransferase (*APRT*) (Table 2). Previously, a study reported [67] the presence of tryptophan-related genes may be associated with IAA production in the bacteria. Similarly, genes related to phosphorus (P), sulfur (S), and nitrogen (N) in CS51 were analyzed (Table 2). Cyanase is an inducible enzyme of *E. coli* found in CS51 (*CynS* and *CynX*), which catalyzes the bicarbonate-dependent decomposition of cyanate. For the N cycle, genes encoding nitrite reductase (*nirB*), nitrate reductase (*napA*), and nitric oxide reductase (*norB*) were detected; these enzymes are involved in the denitrification process, catalyzing the conversion of nitrate to nitrite to nitric oxide, followed by nitric oxide to nitrous oxide [69,70].

Bacteria such as *E. coli* and *B. subtilis* use the phosphate-specific transport (pst) system for the transport of free inorganic phosphate. A previous study [71] has described the structure of the *pst* operon in *E. coli* and *B. subtilis*. The study indicated that the pst operon is composed of *pstS*, *pstC*, *pstA*, and *pstB* as well as a two-component signal transduction system for phosphate uptake consisting of the *phoP/phoR* genes. Based on the analysis of the CS51 complete genome, we found that the pst operon included the *pstA*, *pstB*, *pstC*, and *pstS* genes as well as the *phoB*, *phoP*, and *phoR* genes. CS51 bacteria could regulate the concentration of phosphorus sources via polyphosphate kinases (ppk1 and ppk2) in the extracellular or intracellular environment by polyphosphate degradation and inorganic phosphate transport (*pst* system and *pitA*), which could ensure sufficient inorganic phosphate uptake despite the influence of the extracellular environment. These results are in agreement with the results of another study showing a positive activity for alkaline phosphatase [72,73].

In the genome of CS51, genes related to sulfate transporters (*CysA*, *CysP*, *CysQ*, *CysW*, and *CysT*) were observed (Table 2). Previously, in the superfamily of transport proteins (PiT), *CysP* is the only characterized sulfate transport protein, operated by sulfate: H^+^ symport. Subsequently, the *CysP* gene function was confirmed by expressing *B. subtilis CysP* in *E. coli* with a mutated sulfate transport system [74]. The operon encoded by *CysP* in *B. subtilis* is composed of genes responsible for sulfur metabolism, such as the sulfate adenylyltransferase gene (sat) [75]. *CysP* in *B. subtilis* has been reported to have 10–12 TMHs and homologous domains due to internal gene duplication [74]. 

### 3.6. Phytoremediation Strategies and Resistance to Heavy Metals

Several mechanisms have been reported in the literature regarding the heavy metal stress tolerance of different bacterial species [76]. Analysis of the CS51 genome revealed the presence of several genes involved in the homeostasis of heavy metals. Specifically, the *Cop* operon, which is a membrane cation transporter, was identified. This operon was composed of structural genes, such as the copper resistance genes *CopC*, *CopD*, *CopA*, and *CopB* (Table 3). Similarly, the structure of this copper-inducible operon has been found in different bacterial species such as *P. syringae*, *Xanthomonas campestris*, and *E. coli* [77,78,79,80,81]. The cop operon encodes for proteins responsible for copper sequestration and compartmentalization in the periplasm and outer membrane [82,83]. Furthermore, other copper uptake functions have been associated with this operon [82]. *CopA* encodes for periplasmic proteins, which bind with multiple copper atoms, whereas *CopB* encodes for an outer membrane protein. In a previous study, among different isolated bacteria, 62% of bacterial strains were found to exhibit substantial resistance against copper [84]. Further analysis revealed the presence of cop or cop-like gene systems in 49% of isolates. A subsequent study [85] reported that the main mechanism underlying copper resistance in the plant pathogen *P. syringae* may be attributed to four types of proteins (*CopB*, *CopC*, *CopA*, and *CopD*), which are responsible for copper accumulation in the periplasm and outer membrane. 

Furthermore, the magnesium and cobalt efflux resistance genes *CorA* and *CorC* were found in the CS51 genome (Table 3). These genes have been reported to be involved in Mg^2+^ transport. *CorA* and *MgtE,* which are considered the primary Mg^2+^ transporters in bacteria, have a wide phylogenetic distribution, and the corresponding genes have been reported to be transcribed from constitutive promoters [86,87,88]. The current study revealed the presence of the *cbt*A and *cbtC* genes, which are putatively involved in Zn homeostasis. Previously, the *cbtA* gene was found in *Agrobacterium tumefaciens*, which is thought to be involved in Ni transport. However, the existence of this gene was not predicted in the genome of *S. meliloti* [89]. 

In addition, analysis of the CS51 genome revealed the presence of genes (*CzcD*, *CobW*, *CcmF*, and *CutE*) involved in cobalt-zinc-cadmium resistance and Cu homeostasis (Table 3). Most of the bacterial genes that confer copper resistance are organized in operons [90,91,92]. Various bacterial genes including *copA*, *copB*, *copC*, and *copD* are thought to be involved in Cu resistance [93]. The *copA* gene was identified in the genome of CS51, which encodes multi-copper oxidase, an important copper resistance protein in Gram-negative bacteria [94,95,96]. 

### 3.7. Comparison with Related Species 

**Figure 5:**
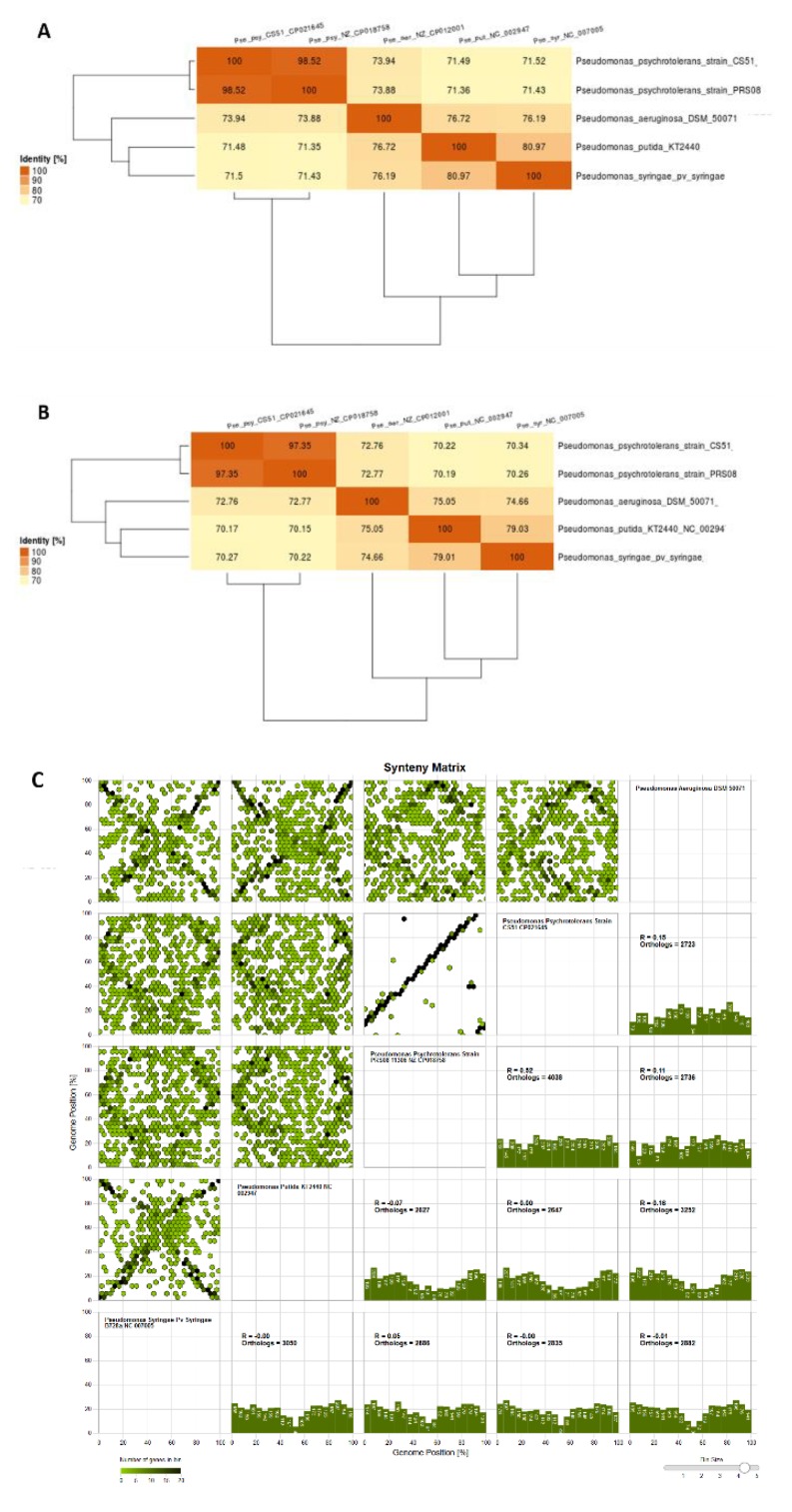
(**A**) Average nucleotide identity (ANI), (**B**) average amino acid identity (AAI) values of *P. psychrotolerans* CS51 and four *Pseudomonas* species, and (**C**) synteny matrix of *P. psychrotolerans* CS51 with related species.

The size of the genome of CS51 was within the expected range (based on related genomes); it was larger than the *P. psychrotolerans* PRSO8 strain and smaller than other related species (Table S1). The GC content was similar to that of *P. psychrotolerans* PRSO8, lower than that of *P. aeruginosa*, and higher than that of *P. putida* and *P. syringae*. In addition, variation was observed in the total number of genes detected in these genomes; a higher number of genes (5,980) were identified in *P. aeruginosa* (Table S1). Furthermore, visual examination of the circular mapping of genomes revealed that the *P. psychrotolerans* PRSO8 strain was more syntenic and showed a higher similarity to CS51; however, it also showed variations in some regions. The other three genomes showed variations in various regions (Figure 4). Whole genome sequencing has delivered new taxonomic metrics, e.g., average nucleotide identity (ANI) and average amino acid identity (AAI), calculated from pairwise comparisons of all sequences shared between any two strains (Figure 5A,B). These are measures of genetic relatedness based on sequences conserved among compared genomes and have gained acceptance as a method for defining bacterial species [97,98]. The results are consistent with circular mapping, and CS51 was more similar to *P. psychrotolerans* PRSO8 with 98.52% ANI and 9.35 % AAI (Figure 5A-B). Furthermore, the synteny plot showed the conservation of the gene order of CS51 with related genomes, and the continuous line in the dot plots of CS51 and *P*. *psychrotolerans* PRSO8 revealed 4,038 orthologs. Many parallel and discontinuous lines were also observed in the dot plots of CS51 (Figure 5C). Genomic rearrangements are important for bacterial evaluation, such as genome reduction processes [99].

**Figure 6:**
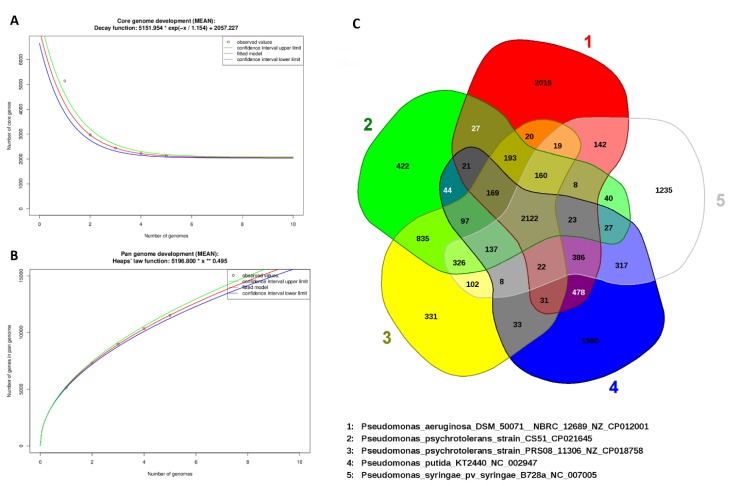
(**A**) The number of gene clusters in the core genome, (**B**) the number of gene clusters in the pan-genome was plotted against the number of *Pseudomonas* species genomes, and (**C**) Venn diagram illustrating the orthologous gene complements of *P. psychrotolerans* CS51, *P. aeruginosa*, *P. psychrotolerans PRS08*, *P. putida*, and *P. syringae.* The number in the center represents orthologous sequences common to all five genomes, whereas the unique genes identified in the genomes are in the outer circles.

A complement of genes in a clade is often defined by the pan-genome. In this study, with a focus on the pan-genome and core genome of the *Pseudomonas* genus, CS51 was sequenced with four other *Pseudomonas* species. The genomes analyzed in this study were plotted against the pan-genome and core genome sizes. When an extra genome was added, the unique gene number, the number of analogous gene clusters that comprise the core genome, was slightly decreased (Figure 6A). However, in the pan-genome, the number of unique genes was increased. Curve extrapolation showed that the core genome was composed of 2122 genes (95% confidence interval = 2034.24 to 2080.215) when the genomes of *P. syringae*, *P. putida*, *P. psychrotolerans* PRS08, and *P. aeruginosa* were added. Owing to duplicated and paralogous genes, the number of shared genes was different in each genome. Moreover, analysis of the pan-genome showed that for each sequenced genome of the *Pseudomonas* species, 1500 new genes (average) were added to the pan-genome (Figure 6B).

Similarly, the curve of the pan-genome showed that the characteristic species of the *Pseudomonas* genus had an open pan-genome. The genome number examined was insufficient for the description of gene sets; thus, to describe all genes in this genus, the sequencing of additional *Sphingomonas* species would be required. In the same species or genus, conserved genes are present in the genome of the bacteria. The core genome contains genes that are conserved, particularly those that are similar and found across the genome in the bacteria. The core genome is often present at both the genus and species level [100], and it is usually used for the identification of variable genes in the genome [101]. Generally, conserved genes are slower to evolve and may be used to determine bacterial associations [102]. The 2122 genes in the Venn diagram are common among all the five species of *Pseudomonas*. The CS51 species shares 27 genes with *P. aeruginosa,* 835 genes with *P. psychrotolerans* PRS08, 44 genes with *P. putida*, and 40 genes with *P. syringae*. On the other hand, there are 422 unique genes in *P. aeruginosa*, 2015 unique genes in *P. psychrotolerans* PRS08, 331 unique genes in *P. putida*, and 1590 unique genes in *P.* syringae (Figure 6C). 

## 4. Conclusions

Genome sequencing of *P*. *psychrotolerans* CS51 has opened up a number of opportunities to study this potential plant growth promoting bacterium in the future. The results of this sequence will benefit the development of a more complete understanding of the mechanisms used by this bacterium to promote plant growth and alleviate heavy metal stress. From the results, it was concluded that CS51 inoculation induced endogenous IAA and GA, to promote plant growth under heavy metal stress. Similarly, genomics analysis revealed that CS51 genome consisted of a circular chromosome of 5,364,174 base pairs having 4774 CDSs genes, 15 rRNA genes, and 67 tRNA genes. Furthermore, the heavy metal resistance of CS51 was confirmed by the detection of cobalt-zinc-cadmium resistant genes in its genome sequence. This work aims to initiate a more comprehensive study and will provide a fundamental basis for future studies towards fully understanding the functioning of this bacterium. Similarly, the availability of the whole genome contents of CS51 will help to provide more insight in unraveling the complex biological mechanisms which indicated that CS51 may be used as an eco-friendly bioresource to promote plant growth in heavy metal-contaminated areas.

## Figures and Tables

**Table 1:** Genomic features, gene prediction, and annotation summary of the CS51 genome.

Annotation Statistics	
Genome size (bp)	5,364,174
GC (%)	64.71
Total number of genes	4859
Number of CDSs	4771
Coding genes	4683
Pseudogenes	88
RNA	88
rRNA (5 S, 16 S, 23 S)	5, 5, 5
rRNA genes	15
tRNA genes	67
ncRNA	6
Protein-coding genes with function prediction	3950
Protein-coding genes without function prediction	733
Protein-coding genes encoding enzymes	1323
Protein coding genes connected to KEGG pathways	1577
Protein coding genes connected to KEGG Orthology (KO)	2726
Protein coding genes connected to MetaCyc pathways	1128
Protein coding genes with COGs	3683

**Table 2:** Genes related to plant growth-promoting activities in the CS51 genome.

Activity Description	Genes in Growth Promoting Activities	Gene Annotation	Gene Names
Plant Hormones	Auxin biosynthesis	Tryptophan synthase alpha chain (Tsa)	*(Tsa)*
	Auxin biosynthesis	Anthranilate phosphoribosyltransferase (APRT)	*(APRT)*
	Auxin biosynthesis	Tryptophan synthase beta chain (TSB)	*(TSB)*
	Auxin biosynthesis	Phosphoribosylanthranilate isomerase (PRAI)	*(PRAI)*
Siderophores	Siderophore Enterobactin	Enterobactin esterase (Fes)	*(Fes)*
	Siderophore Enterobactin	Enterobactin synthetase component F, serine activating enzyme (EC 2.7.7.)	*EntF*
	Siderophore Enterobactin	Enterobactin exporter EntS	*EntS*
	Siderophore Enterobactin	Ferric enterobactin transport system permease protein FepG (TC 3.A.1.14.2)	*FepG*
	Siderophore Enterobactin	Ferric enterobactin-binding periplasmic protein FepB (TC 3.A.1.14.2)	*FepB*
	Siderophore Enterobactin	Ferric enterobactin transport system permease protein FepD (TC 3.A.1.14.2)	*FepD*
	Siderophore Enterobactin	Ferric enterobactin transport ATP-binding protein FepC (TC 3.A.1.14.2)	*FepC*
Nitrogen Metabolism	Cyanate hydrolysis	Cyanate transport protein CynX	*CynX*
	Cyanate hydrolysis	Cyanate hydratase (EC 4.2.1.104)	*CynS*
	Nitrosative stress	Anaerobic nitric oxide reductase transcription regulator NorR	*NorR*
	Amidase clustered with urea and nitrile hydratase functions	Amidase clustered with urea ABC transporter and nitrile hydratase functions	
	Nitrate and nitrite ammonification	Nitrate ABC transporter, ATP-binding protein	
	Nitrate and nitrite ammonification	Response regulator NasT	*NasT*
	Ammonia assimilation	Glutamate synthase [NADPH] putative GlxC chain (EC 1.4.1.13)	*GlxC*
	Ammonia assimilation	Glutamine amidotransferase protein GlxB (EC 2.4.2.-)	*GlxB*
Phosphorus Metabolism	Phosphate metabolism	Phosphate regulon transcriptional regulatory protein PhoB (SphR)	*PhoB*
	Phosphate metabolism	Phosphate transport system permease protein PstC (TC 3.A.1.7.1)	*PstC*
	Phosphate metabolism	Phosphate transport ATP-binding protein PstB (TC 3.A.1.7.1)	*PstB*
	Phosphate metabolism	Phosphate transport system permease protein PstA (TC 3.A.1.7.1)	*PstA*
	Phosphate metabolism	Phosphate transport system regulatory protein PhoU	*PhoU*
	Phosphate metabolism	Predicted ATPase related to phosphate starvation-inducible protein PhoH	*PhoH*
	Phosphate metabolism	Phosphate ABC transporter, periplasmic phosphate-binding protein PstS (TC 3.A.1.7.1)	*PstS*
	Phosphate metabolism	Phosphate starvation-inducible protein PhoH, predicted ATPase	*PhoH*
	Phosphate metabolism	Phosphate regulon sensor protein PhoR (SphS) (EC 2.7.13.3)	*PhoR*
Sulfur Metabolism	Inorganic Sulfur Assimilation	Putative sulfate permease	*Sulp3*
	Inorganic Sulfur Assimilation	Adenylylsulfate kinase (EC 2.7.1.25)	*ASK*
	Inorganic Sulfur Assimilation	Sulfate and thiosulfate binding protein *CysP*	*CysP*
	Inorganic Sulfur Assimilation	3’(2’),5’-bisphosphate nucleotidase (EC 3.1.3.7)	*CysQ*
	Inorganic Sulfur Assimilation	Sulfate transport system permease protein *CysW*	*CysW*
	Inorganic Sulfur Assimilation	Sulfate transport system permease protein *CysT*	*CysT*
	Inorganic Sulfur Assimilation	Sulfate adenylyltransferase subunit 2 (EC 2.7.7.4)	*SAT2*
	Inorganic Sulfur Assimilation	Sulfate adenylyltransferase subunit 1 (EC 2.7.7.4)	*SAT1*
	Inorganic Sulfur Assimilation	Sulfate and thiosulfate import ATP-binding protein *CysA* (EC 3.6.3.25)	*CysA*
	Inorganic Sulfur Assimilation	Ferredoxin	*FDX*
Protein Metabolism	GroEL GroES	Heat shock protein GrpE	*GrpE*
	GroEL GroES	Heat shock protein 60 family co-chaperone GroES	*GroES*
	GroEL GroES	Heat shock protein 60 family chaperone GroEL	*GroEL*
	Protein chaperones	Heat shock protein GrpE	*GrpE*

**Table 3:** Genes potentially involved in heavy metal resistance in the CS51 genome.

Subsystem	Role	Gene Names
Cobalt-zinc-cadmium resistance	Cobalt-zinc-cadmium resistance protein *CzcD*	*CzcD*
Copper homeostasis: copper tolerance	Magnesium and cobalt efflux protein *CorC*	*CorC*
Magnesium transport	Magnesium and cobalt transport protein *CorA*	*CorA*
Magnesium transport	Magnesium and cobalt efflux protein *CorC*	*CorC*
Transport of Nickel and Cobalt	Predicted cobalt transporter *CbtC*	*CbtC*
Transport of Nickel and Cobalt	Predicted cobalt transporter *CbtA*	*CbtA*
G3E family of P-loop GTPases (metallocenter biosynthesis)	GTPase involved in cobalt insertion for B12 biosynthesis	*CobW*
Copper homeostasis	Multicopper oxidase	
Copper homeostasis	Copper resistance protein *CopC*	*CopC*
Copper homeostasis	Multidrug resistance transporter, Bcr/CflA family	
Copper homeostasis	Cu(I)-responsive transcriptional regulator	
Copper homeostasis	Cytochrome c heme lyase subunit *CcmF*	*CcmF*
Copper homeostasis	Copper resistance protein *CopD*	*CopD*
Copper homeostasis	Copper chaperone	
Copper homeostasis	Copper-translocating P-type ATPase (EC 3.6.3.4)	
Copper homeostasis	Copper resistance protein B	*copB*
Copper homeostasis: copper tolerance	Copper homeostasis protein *CutE*	*CutE*
Copper homeostasis: copper tolerance	Magnesium and cobalt efflux protein *CorC*	*CorC*
Copper uptake system *CopCD*	Copper resistance protein *CopD*	*CopD*
Copper uptake system *CopCD*	Copper resistance protein *CopC*	*CopC*
Copper transport system	Copper-translocating P-type ATPase (EC 3.6.3.4)	
Copper transport system	Copper resistance protein *CopC*	*CopC*
Copper transport and blue copper proteins	Azurin	
Copper homeostasis	Copper resistance protein A	*CopA*

## Supplementary Materials

The following are available online at https://www.mdpi.com/2076-2607/8/3/382/s1.
