# Supplementary material for: Complete Genome Sequence of Pseudomonas psychrotolerans CS51, a Plant Growth-Promoting Bacterium, Under Heavy Metal Stress Conditions
Source: Microorganisms. 2020 Mar 9;8(3):382. doi: 10.3390/microorganisms8030382 (PMC7142416; doi:10.3390/microorganisms8030382)
Supplement: Supplementary file 1 [file microorganisms-08-00382-s001.pdf]

# Supplementary Data

| Name | Count | Percent |
|---|---|---|
| Amino acid transport and metabolism | 456 | 10.93% |
| Carbohydrate transport and metabolism | 288 | 6.9% |
| Cell cycle control, cell division, chromosome partitioning | 39 | 0.93% |
| Cell motility | 153 | 3.67% |
| Cell wall/membrane/envelope biogenesis | 254 | 6.09% |
| Chromatin structure and dynamics | 1 | 0.02% |
| Coenzyme transport and metabolism | 219 | 5.25% |
| Cytoskeleton | 1 | 0.02% |
| Defense mechanisms | 75 | 1.8% |
| Energy production and conversion | 249 | 5.97% |
| Extracellular structures | 27 | 0.65% |
| Function unknown | 217 | 5.2% |
| General function prediction only | 333 | 7.98% |
| Inorganic ion transport and metabolism | 264 | 6.33% |
| Intracellular trafficking, secretion, and vesicular transport | 60 | 1.44% |
| Lipid transport and metabolism | 174 | 4.17% |
| Mobilome: prophages, transposons | 47 | 1.13% |
| Nucleotide transport and metabolism | 100 | 2.4% |
| Posttranslational modification, protein turnover, chaperones | 144 | 3.45% |
| RNA processing and modification | 1 | 0.02% |
| Replication, recombination and repair | 123 | 2.95% |
| Secondary metabolites biosynthesis, transport and catabolism | 96 | 2.3% |
| Signal transduction mechanisms | 280 | 6.71% |
| Transcription | 333 | 7.98% |
| Translation, ribosomal structure and biogenesis | 239 | 5.73% |
| Not in COG | 1087 | 22.79% |

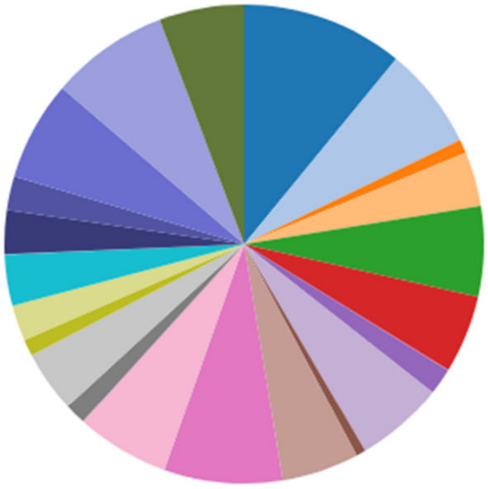

**Figure S1.** Genes (number and percentage) in the CS51 genome related to COG categories.

KEGG Categories | Statistics by KEGG categories

| Name | Count | Percent |
|---|---|---|
| Aging | 9 | 0.28% |
| Amino acid metabolism | 275 | 8.66% |
| Biosynthesis of other secondary metabolites | 37 | 1.17% |
| Cancers: Overview | 27 | 0.85% |
| Cancers: Specific types | 4 | 0.13% |
| Carbohydrate metabolism | 270 | 8.51% |
| Cardiovascular diseases | 1 | 0.03% |
| Cell growth and death | 19 | 0.6% |
| Cell motility | 108 | 3.4% |
| Chemical structure transformation maps | 1 | 0.03% |
| Circulatory system | 3 | 0.09% |
| Digestive system | 1 | 0.03% |
| Drug resistance | 46 | 1.45% |
| Endocrine and metabolic diseases | 10 | 0.32% |
| Endocrine system | 14 | 0.44% |
| Energy metabolism | 175 | 5.51% |
| Environmental adaptation | 7 | 0.22% |
| Excretory system | 1 | 0.03% |
| Folding, sorting and degradation | 50 | 1.58% |
| Global and overview maps | 830 | 26.15% |
| Glycan biosynthesis and metabolism | 44 | 1.39% |
| Immune diseases | 2 | 0.06% |
| Immune system | 2 | 0.06% |
| Infectious diseases: Bacterial | 27 | 0.85% |
| Infectious diseases: Parasitic | 3 | 0.09% |
| Infectious diseases: Viral | 1 | 0.03% |
| Lipid metabolism | 75 | 2.36% |
| Membrane transport | 252 | 7.94% |
| Metabolism of cofactors and vitamins | 176 | 5.55% |
| Metabolism of other amino acids | 73 | 2.3% |
| Metabolism of terpenoids and polyketides | 62 | 1.95% |
| Nervous system | 7 | 0.22% |
| Neurodegenerative diseases | 12 | 0.38% |
| Nucleotide metabolism | 111 | 3.5% |
| Replication and repair | 51 | 1.61% |
| Signal transduction | 194 | 6.11% |
| Substance dependence | 1 | 0.03% |
| Transcription | 4 | 0.13% |
| Translation | 89 | 2.8% |
| Transport and catabolism | 10 | 0.32% |
| Xenobiotics biodegradation and metabolism | 90 | 2.84% |

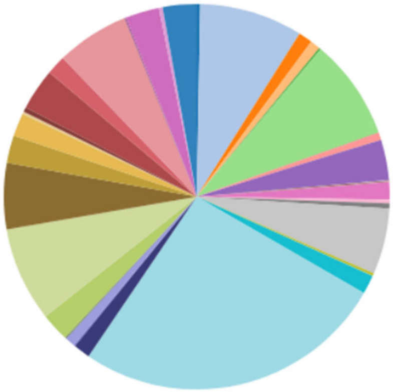

**Figure S2.** Genes (number and percentage) in the CS51 genome related to KEGG categories.

**Table S1.** Genomic comparison of *P. psychrotolerans* CS51 with related species.

| **Annotation statistics** | ***P. psychrotolerans* CS51** | ***P. psychrotolerans* PRSO8** | ***P. aeruginosa*** | ***P. putida*** | ***P.syringae* pv. *Syringae*** |
|---|---|---|---|---|---|
| **Genome size (bp)** | 5,364,174 | 5,271,920 | 6,317,050 | 6,181,873 | 6,093,698 |
| **GC (%)** | 64.71 | 64.8 | 66.5 | 61.5 | 59.2 |
| **Total number of genes** | 4,859 | 4,753 | 5,980 | 5,786 | 5,220 |
| **Number of CDSs** | 4,771 | 4,657 | 5,980 | 5,564 | 5,089 |
| **Pseudogenes** | 88 | NA | 54 | NA | NA |
| **RNA** | 88 | 96 | 80 | 22 | 16 |
| **rRNA (5 S, 16 S, 23 S)** | 5, 5, 5 | 5,5,5 | 4, 4, 4, | NA | NA |
| **rRNA genes** | 15 | 15 | 12 | NA | NA |
| **tRNA genes** | 67 | 68 | 64 | 75 | 64 |
